# Morpholinium 4-amino-5-meth­oxy-2-methyl­benzensulfonate

**DOI:** 10.1107/S1600536811003722

**Published:** 2011-02-12

**Authors:** Ling-Gao Shou, Mei-Chao Li

**Affiliations:** a College of Chemical Engineering and Material Sciences, Zhejiang University of Technology, Hangzhou 310014, People’s Republic of China

## Abstract

In the crystal structure of the title compound, C_4_H_10_NO^+^·C_8_H_10_NO_4_S^−^, the components are linked by N—H⋯O hydrogen bonds, forming a centrosymmetric 2:2 aggregate. The aggregates are further connected by N—H⋯O hydrogen bonds between the anions, forming a double-tape structure along the *a* axis.

## Related literature

For related structures, see: Barbour *et al.* (1996 ▶); Brito *et al.* (2004 ▶); Yin *et al.* (2006 ▶).
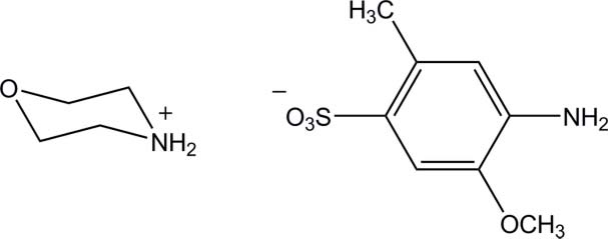


## Experimental

### 

#### Crystal data

C_4_H_10_NO^+^·C_8_H_10_NO_4_S^−^
*M* *_r_* = 304.37
Monoclinic, 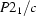

*a* = 9.2141 (5) Å
*b* = 14.8227 (9) Å
*c* = 10.4740 (7) Å
β = 91.120 (2)°
*V* = 1430.24 (15) Å^3^
*Z* = 4
Mo *K*α radiation
μ = 0.25 mm^−1^
*T* = 293 K
0.34 × 0.25 × 0.24 mm

#### Data collection

Bruker SMART APEX CCD diffractometer
Absorption correction: multi-scan (*SADABS*; Bruker, 2001 ▶) *T*_min_ = 0.910, *T*_max_ = 0.931
13039 measured reflections
3105 independent reflections
2962 reflections with *I* > 2σ(*I*)
*R*_int_ = 0.034

#### Refinement

*R*[*F* ^2^ > 2σ(*F* ^2^)] = 0.044
*wR*(*F* ^2^) = 0.117
*S* = 1.12
3105 reflections
191 parameters
H atoms treated by a mixture of independent and constrained refinement
Δρ_max_ = 0.27 e Å^−3^
Δρ_min_ = −0.52 e Å^−3^

### 

Data collection: *SMART* (Bruker, 2007 ▶); cell refinement: *SAINT* (Bruker, 2007 ▶); data reduction: *SAINT*; program(s) used to solve structure: *SHELXS97* (Sheldrick, 2008 ▶); program(s) used to refine structure: *SHELXL97* (Sheldrick, 2008 ▶); molecular graphics: *SHELXTL* (Sheldrick, 2008 ▶); software used to prepare material for publication: *SHELXL97*.

## Supplementary Material

Crystal structure: contains datablocks global, I. DOI: 10.1107/S1600536811003722/is2665sup1.cif

Structure factors: contains datablocks I. DOI: 10.1107/S1600536811003722/is2665Isup2.hkl

Additional supplementary materials:  crystallographic information; 3D view; checkCIF report

## Figures and Tables

**Table 1:** Hydrogen-bond geometry (Å, °)

*D*—H⋯*A*	*D*—H	H⋯*A*	*D*⋯*A*	*D*—H⋯*A*
N1—H1*A*⋯O2^i^	0.90	1.92	2.795 (3)	162
N1—H1*B*⋯O2	0.90	2.56	3.126 (2)	121
N1—H1*B*⋯O3	0.90	1.89	2.790 (3)	175
N2—H2*A*⋯O1^ii^	0.86 (2)	2.23 (2)	3.054 (3)	159.1 (2)
N2—H2*B*⋯O5^iii^	0.85 (2)	2.25 (2)	3.077 (4)	161.5 (2)

Symmetry codes: (i) 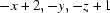; (ii) 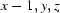; (iii) 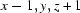.

**Fig. 1.**
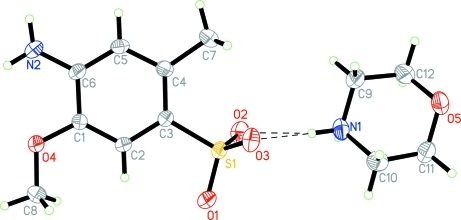
The molecular structure of the title compound, showing the atom-numbering scheme and 50% probability displacement ellipsoids for non-H atoms. Hydrogen bond is illustrated as dashed lines.

**Fig. 2.**
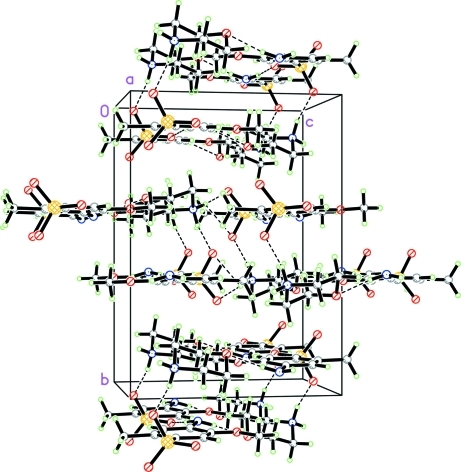
The crystal packing of the title compound, viewed down the a axis. Hydrogen bonds are drawn as dashed lines.

## supplementary crystallographic information

### Comment

Several supramolecular structures of morpholinium sulfonate have been reported previously (Barbour *et al.*, 1996; Yin *et al.*, 2006; Brito *et al.*, 2004). As an extension of research, we report here the structure of the title compound, (I).

As shown in Figs. 1 and 2, the 4-amino-5-methoxy-2-methylbenzensulfonate anion is linked to the morpholinium cation by N1—H1B···O3, N1—H1B···O2 and N1—H1A···O2^i^ hydrogen bonds (Table 1). Bond lengths of morpholine ring are very similar to those observed previously (Barbour *et al.*, 1996; Yin *et al.*, 2006; Brito *et al.*, 2004). N2—H2A···O1^ii^ and N2—H2B···O5^iii^ hydrogen bonds (Table 1) also play important roles in stabilizing the crystal.

### Experimental

4-Amino-5-methoxy-2-methylbenzensulfonic acid (2.2 g) and morpholine (0.9 g), in a molar ratio of 1:1, were mixed and dissolved in sufficient ethanol by heating to 373 K, at which point a clear solution resulted. The system was then cooled slowly to room temperature. Crystals (2.5 g) were formed, collected and washed with ethanol.

### Refinement

H atoms attached to atom N2 were located in a difference Fourier map, and were refined freely. H atoms attached to atom N1 were treated as riding (N—H = 0.90 Å), with *U*_iso_(H) = 1.2*U*_eq_(N). Other H atoms were placed in calculated positions (C—H = 0.93–0.97 Å), with *U*_iso_(H) = 1.2 or 1.5*U*_eq_(C).

### Crystal data

**Table:**

C_4_H_10_NO^+^·C_8_H_10_NO_4_S^−^	*F*(000) = 648.0
*M**_r_* = 304.37	*D*_x_ = 1.413 Mg m^−^^3^
Monoclinic, *P*2_1_/*c*	Melting point: 467 K
Hall symbol: -P 2ybc	Mo *K*α radiation, λ = 0.71073 Å
*a* = 9.2141 (5) Å	Cell parameters from 3615 reflections
*b* = 14.8227 (9) Å	θ = 2.3–25.3°
*c* = 10.4740 (7) Å	µ = 0.25 mm^−^^1^
β = 91.120 (2)°	*T* = 293 K
*V* = 1430.24 (15) Å^3^	Block, colorless
*Z* = 4	0.34 × 0.25 × 0.24 mm

### Data collection

**Table:**

Bruker SMART APEX CCD diffractometer	3105 independent reflections
Radiation source: fine-focus sealed tube	2962 reflections with *I* > 2σ(*I*)
graphite	*R*_int_ = 0.034
φ and ω scans	θ_max_ = 27.0°, θ_min_ = 3.2°
Absorption correction: multi-scan (*SADABS*; Bruker, 2001)	*h* = −11→11
*T*_min_ = 0.910, *T*_max_ = 0.931	*k* = −18→18
13039 measured reflections	*l* = −13→13

### Refinement

**Table:**

Refinement on *F*^2^	Secondary atom site location: difference Fourier map
Least-squares matrix: full	Hydrogen site location: inferred from neighbouring sites
*R*[*F*^2^ > 2σ(*F*^2^)] = 0.044	H atoms treated by a mixture of independent and constrained refinement
*wR*(*F*^2^) = 0.117	*w* = 1/[σ^2^(*F*_o_^2^) + (0.0661*P*)^2^ + 0.3546*P*] where *P* = (*F*_o_^2^ + 2*F*_c_^2^)/3
*S* = 1.12	(Δ/σ)_max_ < 0.001
3105 reflections	Δρ_max_ = 0.27 e Å^−^^3^
191 parameters	Δρ_min_ = −0.52 e Å^−^^3^
0 restraints	Extinction correction: *SHELXL97* (Sheldrick, 2008), Fc^*^=kFc[1+0.001xFc^2^λ^3^/sin(2θ)]^-1/4^
Primary atom site location: structure-invariant direct methods	Extinction coefficient: 0.083 (5)

### Special details

**Table:**

Geometry. All e.s.d.'s (except the e.s.d. in the dihedral angle between two l.s. planes) are estimated using the full covariance matrix. The cell e.s.d.'s are taken into account individually in the estimation of e.s.d.'s in distances, angles and torsion angles; correlations between e.s.d.'s in cell parameters are only used when they are defined by crystal symmetry. An approximate (isotropic) treatment of cell e.s.d.'s is used for estimating e.s.d.'s involving l.s. planes.
Refinement. Refinement of *F*^2^ against ALL reflections. The weighted *R*-factor *wR* and goodness of fit *S* are based on *F*^2^, conventional *R*-factors *R* are based on *F*, with *F* set to zero for negative *F*^2^. The threshold expression of *F*^2^ > σ(*F*^2^) is used only for calculating *R*-factors(gt) *etc*. and is not relevant to the choice of reflections for refinement. *R*-factors based on *F*^2^ are statistically about twice as large as those based on *F*, and *R*- factors based on ALL data will be even larger.

### Fractional atomic coordinates and isotropic or equivalent isotropic displacement parameters (Å^2^)

**Table:**

	*x*	*y*	*z*	*U*_iso_*/*U*_eq_	
H2A	0.152 (2)	0.1046 (13)	0.8007 (19)	0.044 (5)*	
H2B	0.219 (2)	0.1123 (13)	0.925 (2)	0.052 (6)*	
C1	0.48589 (15)	0.11584 (9)	0.87597 (13)	0.0312 (3)	
C2	0.62334 (14)	0.11986 (9)	0.82610 (13)	0.0316 (3)	
H2	0.7039	0.1236	0.8808	0.038*	
C3	0.64240 (14)	0.11834 (8)	0.69411 (13)	0.0290 (3)	
C4	0.52301 (15)	0.11504 (9)	0.61017 (13)	0.0316 (3)	
C5	0.38583 (15)	0.10920 (10)	0.66357 (14)	0.0343 (3)	
H5	0.3052	0.1060	0.6090	0.041*	
C6	0.36395 (14)	0.10799 (9)	0.79442 (14)	0.0307 (3)	
C7	0.53473 (19)	0.11758 (12)	0.46690 (14)	0.0450 (4)	
H7A	0.4465	0.0951	0.4284	0.067*	
H7B	0.6147	0.0806	0.4414	0.067*	
H7C	0.5505	0.1786	0.4397	0.067*	
C8	0.57504 (19)	0.12216 (13)	1.09045 (15)	0.0467 (4)	
H8C	0.5405	0.1230	1.1763	0.070*	
H8D	0.6294	0.1761	1.0748	0.070*	
H8E	0.6363	0.0705	1.0790	0.070*	
C9	0.91130 (17)	0.12103 (11)	0.23520 (16)	0.0427 (4)	
H9A	0.8646	0.1790	0.2222	0.051*	
H9B	0.8363	0.0758	0.2453	0.051*	
C10	1.12875 (18)	0.18648 (12)	0.33325 (16)	0.0462 (4)	
H10A	1.1928	0.1849	0.4078	0.055*	
H10B	1.0931	0.2477	0.3228	0.055*	
C11	1.21090 (17)	0.15881 (13)	0.21691 (16)	0.0471 (4)	
H11A	1.2914	0.1999	0.2047	0.056*	
H11B	1.2502	0.0987	0.2293	0.056*	
C12	1.0018 (2)	0.09834 (14)	0.12131 (16)	0.0526 (4)	
H12A	1.0400	0.0377	0.1310	0.063*	
H12B	0.9408	0.0996	0.0448	0.063*	
O1	0.91647 (12)	0.12197 (9)	0.75110 (12)	0.0501 (3)	
O2	0.83977 (12)	0.02929 (7)	0.57210 (11)	0.0449 (3)	
O3	0.83980 (11)	0.19031 (7)	0.55105 (10)	0.0416 (3)	
O5	1.11893 (13)	0.15976 (10)	0.10715 (11)	0.0533 (3)	
O4	0.45499 (12)	0.11751 (9)	1.00344 (10)	0.0447 (3)	
N1	1.00509 (15)	0.12414 (9)	0.35140 (12)	0.0383 (3)	
H1A	1.0389	0.0685	0.3690	0.046*	
H1B	0.9527	0.1427	0.4181	0.046*	
N2	0.22873 (14)	0.09542 (10)	0.84692 (15)	0.0416 (3)	
S1	0.82261 (3)	0.11518 (2)	0.63924 (3)	0.03170 (15)	

### Atomic displacement parameters (Å^2^)

**Table:**

	*U*^11^	*U*^22^	*U*^33^	*U*^12^	*U*^13^	*U*^23^
C1	0.0266 (7)	0.0409 (7)	0.0263 (6)	0.0017 (5)	0.0007 (5)	−0.0003 (5)
C2	0.0234 (6)	0.0425 (7)	0.0287 (6)	0.0004 (5)	−0.0023 (5)	0.0000 (5)
C3	0.0247 (6)	0.0331 (6)	0.0292 (6)	0.0013 (4)	0.0016 (5)	−0.0007 (5)
C4	0.0310 (7)	0.0359 (7)	0.0278 (6)	0.0026 (5)	−0.0022 (5)	−0.0020 (5)
C5	0.0261 (6)	0.0428 (8)	0.0337 (7)	0.0015 (5)	−0.0068 (5)	−0.0025 (5)
C6	0.0240 (6)	0.0330 (6)	0.0351 (7)	0.0022 (5)	−0.0005 (5)	−0.0005 (5)
C7	0.0442 (9)	0.0628 (10)	0.0279 (7)	0.0011 (7)	−0.0031 (6)	−0.0029 (6)
C8	0.0391 (8)	0.0722 (11)	0.0285 (7)	0.0002 (7)	−0.0041 (6)	0.0019 (7)
C9	0.0326 (8)	0.0515 (9)	0.0441 (9)	0.0013 (6)	0.0020 (6)	0.0035 (6)
C10	0.0465 (9)	0.0476 (9)	0.0444 (8)	−0.0033 (7)	−0.0013 (7)	−0.0057 (7)
C11	0.0357 (7)	0.0563 (10)	0.0495 (9)	−0.0041 (7)	0.0074 (7)	0.0044 (7)
C12	0.0480 (10)	0.0733 (12)	0.0366 (8)	−0.0010 (8)	0.0003 (7)	−0.0092 (8)
O1	0.0255 (5)	0.0828 (9)	0.0420 (6)	−0.0007 (5)	−0.0006 (5)	0.0016 (5)
O2	0.0445 (6)	0.0391 (6)	0.0515 (6)	0.0111 (4)	0.0101 (5)	−0.0029 (5)
O3	0.0421 (6)	0.0389 (6)	0.0442 (6)	−0.0001 (4)	0.0136 (5)	0.0033 (4)
O5	0.0477 (7)	0.0754 (9)	0.0371 (6)	0.0013 (6)	0.0099 (5)	0.0109 (6)
O4	0.0280 (5)	0.0799 (9)	0.0263 (5)	0.0004 (5)	0.0018 (4)	0.0003 (5)
N1	0.0404 (7)	0.0415 (7)	0.0336 (6)	0.0080 (5)	0.0096 (5)	0.0055 (5)
N2	0.0233 (6)	0.0583 (8)	0.0431 (7)	0.0010 (5)	0.0007 (5)	−0.0006 (6)
S1	0.0252 (2)	0.0379 (2)	0.0322 (2)	0.00271 (11)	0.00416 (14)	0.00068 (12)

### Geometric parameters (Å, °)

**Table:**

C1—O4	1.3707 (17)	C9—C12	1.507 (2)
C1—C2	1.3806 (19)	C9—H9A	0.9700
C1—C6	1.4029 (19)	C9—H9B	0.9700
C2—C3	1.3972 (19)	C10—N1	1.482 (2)
C2—H2	0.9300	C10—C11	1.504 (2)
C3—C4	1.3955 (19)	C10—H10A	0.9700
C3—S1	1.7683 (13)	C10—H10B	0.9700
C4—C5	1.395 (2)	C11—O5	1.415 (2)
C4—C7	1.507 (2)	C11—H11A	0.9700
C5—C6	1.389 (2)	C11—H11B	0.9700
C5—H5	0.9300	C12—O5	1.422 (2)
C6—N2	1.3842 (18)	C12—H12A	0.9700
C7—H7A	0.9600	C12—H12B	0.9700
C7—H7B	0.9600	O1—S1	1.4461 (12)
C7—H7C	0.9600	O2—S1	1.4645 (11)
C8—O4	1.4211 (18)	O3—S1	1.4575 (11)
C8—H8C	0.9600	N1—H1A	0.9000
C8—H8D	0.9600	N1—H1B	0.9000
C8—H8E	0.9600	N2—H2A	0.86 (2)
C9—N1	1.480 (2)	N2—H2B	0.86 (2)
			
O4—C1—C2	125.26 (12)	N1—C10—C11	109.53 (13)
O4—C1—C6	114.54 (12)	N1—C10—H10A	109.8
C2—C1—C6	120.19 (12)	C11—C10—H10A	109.8
C1—C2—C3	120.51 (12)	N1—C10—H10B	109.8
C1—C2—H2	119.7	C11—C10—H10B	109.8
C3—C2—H2	119.7	H10A—C10—H10B	108.2
C4—C3—C2	120.74 (12)	O5—C11—C10	110.65 (13)
C4—C3—S1	121.87 (11)	O5—C11—H11A	109.5
C2—C3—S1	117.32 (10)	C10—C11—H11A	109.5
C5—C4—C3	117.32 (12)	O5—C11—H11B	109.5
C5—C4—C7	118.94 (13)	C10—C11—H11B	109.5
C3—C4—C7	123.74 (13)	H11A—C11—H11B	108.1
C6—C5—C4	123.13 (12)	O5—C12—C9	111.88 (15)
C6—C5—H5	118.4	O5—C12—H12A	109.2
C4—C5—H5	118.4	C9—C12—H12A	109.2
N2—C6—C5	122.87 (13)	O5—C12—H12B	109.2
N2—C6—C1	119.06 (13)	C9—C12—H12B	109.2
C5—C6—C1	118.01 (13)	H12A—C12—H12B	107.9
C4—C7—H7A	109.5	C11—O5—C12	110.65 (12)
C4—C7—H7B	109.5	C1—O4—C8	116.84 (12)
H7A—C7—H7B	109.5	C9—N1—C10	110.61 (12)
C4—C7—H7C	109.5	C9—N1—H1A	109.5
H7A—C7—H7C	109.5	C10—N1—H1A	109.5
H7B—C7—H7C	109.5	C9—N1—H1B	109.5
O4—C8—H8C	109.5	C10—N1—H1B	109.5
O4—C8—H8D	109.5	H1A—N1—H1B	108.1
H8C—C8—H8D	109.5	C6—N2—H2A	119.5 (13)
O4—C8—H8E	109.5	C6—N2—H2B	116.8 (15)
H8C—C8—H8E	109.5	H2A—N2—H2B	112.7 (19)
H8D—C8—H8E	109.5	O1—S1—O3	112.93 (7)
N1—C9—C12	109.57 (13)	O1—S1—O2	112.40 (7)
N1—C9—H9A	109.8	O3—S1—O2	110.23 (7)
C12—C9—H9A	109.8	O1—S1—C3	106.58 (7)
N1—C9—H9B	109.8	O3—S1—C3	107.47 (6)
C12—C9—H9B	109.8	O2—S1—C3	106.86 (6)
H9A—C9—H9B	108.2		

### Hydrogen-bond geometry (Å, °)

**Table:**

*D*—H···*A*	*D*—H	H···*A*	*D*···*A*	*D*—H···*A*
N1—H1A···O2^i^	0.90	1.92	2.795 (3)	162
N1—H1B···O2	0.90	2.56	3.126 (2)	121
N1—H1B···O3	0.90	1.89	2.790 (3)	175
N2—H2A···O1^ii^	0.86 (2)	2.23 (2)	3.054 (3)	159.1 (2)
N2—H2B···O5^iii^	0.85 (2)	2.25 (2)	3.077 (4)	161.5 (2)

Symmetry codes: (i) −*x*+2, −*y*, −*z*+1; (ii) *x*−1, *y*, *z*; (iii) *x*−1, *y*, *z*+1.
